# A Small-Scale Comparison of Iceland Scallop Size Distributions Obtained from a Camera Based Autonomous Underwater Vehicle and Dredge Survey

**DOI:** 10.1371/journal.pone.0109369

**Published:** 2014-10-10

**Authors:** Warsha Singh, Erla B. Örnólfsdóttir, Gunnar Stefansson

**Affiliations:** 1 Science Institute, University of Iceland, Reykjavik, Iceland; 2 Holar University College, Holar, Iceland; 3 Science Institute, University of Iceland, Reykjavik, Iceland; Ocean University of China, China

## Abstract

An approach is developed to estimate size of Iceland scallop shells from AUV photos. A small-scale camera based AUV survey of Iceland scallops was conducted at a defined site off West Iceland. Prior to height estimation of the identified shells, the distortions introduced by the vehicle orientation and the camera lens were corrected. The average AUV pitch and roll was 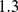 and 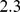 deg that resulted in 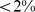 error in ground distance rendering these effects negligible. A quadratic polynomial model was identified for lens distortion correction. This model successfully predicted a theoretical grid from a frame photographed underwater, representing the inherent lens distortion. The predicted shell heights were scaled for the distance from the bottom at which the photos were taken. This approach was validated by height estimation of scallops of known sizes. An underestimation of approximately 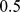 cm was seen, which could be attributed to pixel error, where each pixel represented 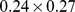 cm. After correcting for this difference the estimated heights ranged from 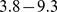 cm. A comparison of the height-distribution from a small-scale dredge survey carried out in the vicinity showed non-overlapping peaks in size distribution, with scallops of a broader size range visible in the AUV survey. Further investigations are necessary to evaluate any underlying bias and to validate how representative these surveys are of the true population. The low resolution images made identification of smaller scallops difficult. Overall, the observations of very few small scallops in both surveys could be attributed to low recruitment levels in the recent years due to the known scallop parasite outbreak in the region.

## Introduction

Length frequency distributions form the most basic data source for investigating the population dynamics of a species. In its simplest form a size distribution may provide a description of a recruitment signal and a few years of such data may indicate whether recruitment is variable or stable, whether growth is fast or slow, and thus provide indications on the longevity of the species. They are also particularly useful when age data are difficult to gather. The Iceland scallop (*Chlamys islandica* (O.F. Müller)) fishery in Iceland has so far been monitored with the use of the conventional dredge survey technique. Numerous studies have shown this method to be size selective and lacking in capture efficiency [1], [2], which can lead to conservative population estimates and misrepresentations in the underlying population structure [3]. The negative impact it has on the habitat structure of the macrobenthic organisms also makes this technique unpopular [4], [5]. A shift away from this traditional approach is to estimate abundance and size distributions from photographs and video surveys of the seafloor [6]–[8]. A comparative survey between dredge and video surveys of sea scallops conducted by [9] concluded that video surveys gave better estimates of shell height frequency distribution and densities.

Towed and tethered camera systems are dependent on a research vessel for operation, which increases their operational costs. In comparison, the capability of an autonomous underwater vehicle (AUV) to operate independently of a research vessel makes this survey technique potentially more cost effective [10]. AUVs can be deployed from any type of research vessel irrespective of their size, or simply deployed from the shore with minimal manpower. Shallow water habitats can also be surveyed where research vessels cannot operate. It is also more feasible to take sufficient replicates in a given area with this method. Repeating entire surveys to assess the confidence level of key population parameters such as density estimates is also possible, as shown by [11]. Some of the drawbacks of the AUV survey technique include limited battery power, little communication with the vehicle operator while in mission, and the need to surface up to obtain a correct geographic position fix via GPS. An Acoustic Doppler Current Profiler (ADCP), compass, and Inertial Navigation System (INS) are used to track position while submerged.

AUV photos have been used in the past to estimate lengths of groundfish by [12]. Fish size estimates were then related to the different substrate types and a logistic regression curve was used to determine the maturity of observed individuals. In a recent study by [10] an AUV based stereo-camera was used to assess the stocks of Ocean Perch *Helicolenus percoides* and estimate the lengths of individual fish to assess the length frequency distributions. Shell height estimation of scallop shells from video surveys has been done previously by [6], [7], [13], [14] among others. Studies using towed camera systems include [15]. High resolution imagery has also been used to detect juvenile scallops of size 10 mm [16].

The scallop fishery in Iceland commenced in 1969. A combined effect of protozoan infestation and scallop dredging caused a steep descent in the stock index from 2000 to 2003, which led to a closure of the fishery in 2004 [17], [18]. To develop the use of an AUV as a stock assessment tool is worthwhile for fragile habitats such as the current sampling grounds. In this study, a small AUV survey was carried out to study scallop populations in a defined site off West Iceland. Methods were developed for size estimation of individual scallop shells from the AUV photos. Repeated measurements were used to generate a confidence interval around the observed size distribution. A small-scale comparison was then carried out between the shell height frequency distributions obtained from the AUV survey and available data from nearby dredge sampling sites.

## Materials and Methods

### AUV Survey

**Figure 1:**
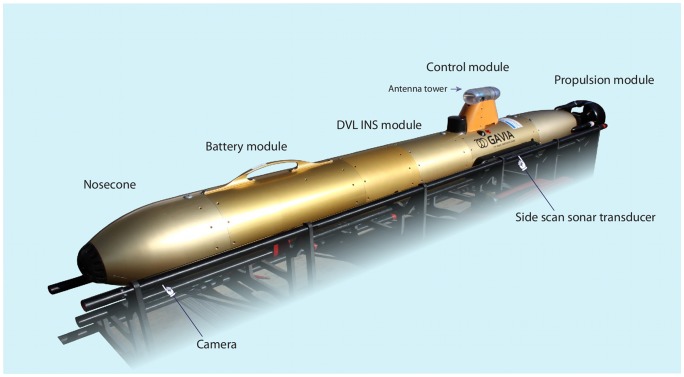
The Gavia autonomous underwater vehicle used for this study. Modules include nosecone with camera, battery module, doppler velocity log, control unit with tower and side-scan sonar, and propulsion module. The strobe is located under the control module.

A Gavia AUV (Figure 1) was used to sample a known scallop ground approximately 3 km north of Stykkisholmur, West Iceland (65°05.598′ N 22°45.500′ W) in November 2011. No specific permits were required for field work because this was an academic study and a collaborative work between University of Iceland and the Vör Marine Research Center at Breidafjordur, West Iceland. The survey region is not privately owned or classified as a protected area, and sampling did not involve endangered or protected species. The sampling location is close to an area monitored by the Marine Research Institute of Iceland on a regular basis through dredge surveys. A small-scale experimental survey was conducted that composed of 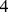 parallel lines of 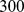 m in length spaced 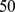 m apart. This was repeated 5 times within the survey location for variance estimation. This survey was first described in [11] who analyzed the survey for repeatability and for scallop abundance measurements. The survey description is repeated here for completeness.

The AUV was navigated at 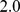 m from the bottom at 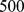 rpm that roughly corresponds to 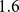 m/sec. The starting point of each survey was chosen at random. The planned mission time for each survey was approximately 20 min. Both digital photographs and side-scan sonar data were collected during the survey. The water depth at the sampling location ranged between 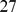 and 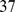 m. Four transects of approximately 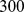 m in length were extracted from each survey for analysis. Given the imprecise positioning information of this particular Gavia, the data were simply considered to be samples from possible transects within the survey area. Depth variations in the vehicle path caused the distance from the bottom at which the photographs were taken (altitude) to range from 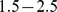 m. Given that the sea floor in the survey area is rather flat, these depth variations could be related to the strong current flow against which the vehicle had to maneuver, making it difficult to maintain a constant altitude from the bottom. Strong tidal currents can also lead to murky water conditions, in turn, affecting the visibility in the images. Images taken at greater distances from the bottom >2.5 m) cover more area on the seabed but tend to lack details. Identification of smaller scallops can also be more challenging with a wider field of view. Thus, for clear visibility, a subset of images taken at 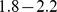 m from the bottom was extracted from the data set. The percentage of photographs that comprised this subset was dependent on the steadiness of the vehicle along the different transects. For instance, in survey 4 transect 2, 97% of the photos were taken within the defined range whereas in transect 3 only 40% were within 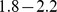 m. A random sample of 25 images per transect were selected from this subset, which gave 100 samples in total per survey. The random samples of photographs were thus considered to be representative of the population in the area. A random selection was applied to avoid any bias in image selection.

The camera on the AUV is a Scorpion model SCOR-20CSO color digital camera with a resolution 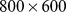. The camera is located on the underside of the nose module and the lighting comes from a strobe comprised of 20 LEDs located on the underside of the control module. The camera was configured to take 3 frames/sec that ensures 50% overlap between the images [19]. The photos were stored in an 8-bit jpeg format. Each photo contains embedded metadata on time, GPS location, altitude (distance from the bottom), depth (distance from the surface), pitch (movement of the AUV along the y-axis causing a upward or downward tilt), roll (movement along the x-axis causing a sideways right or left tilt), and image properties such as brightness, exposure, gamma, white-balance, and shutter speed.

### Shell identification & size estimation

**Figure 2:**
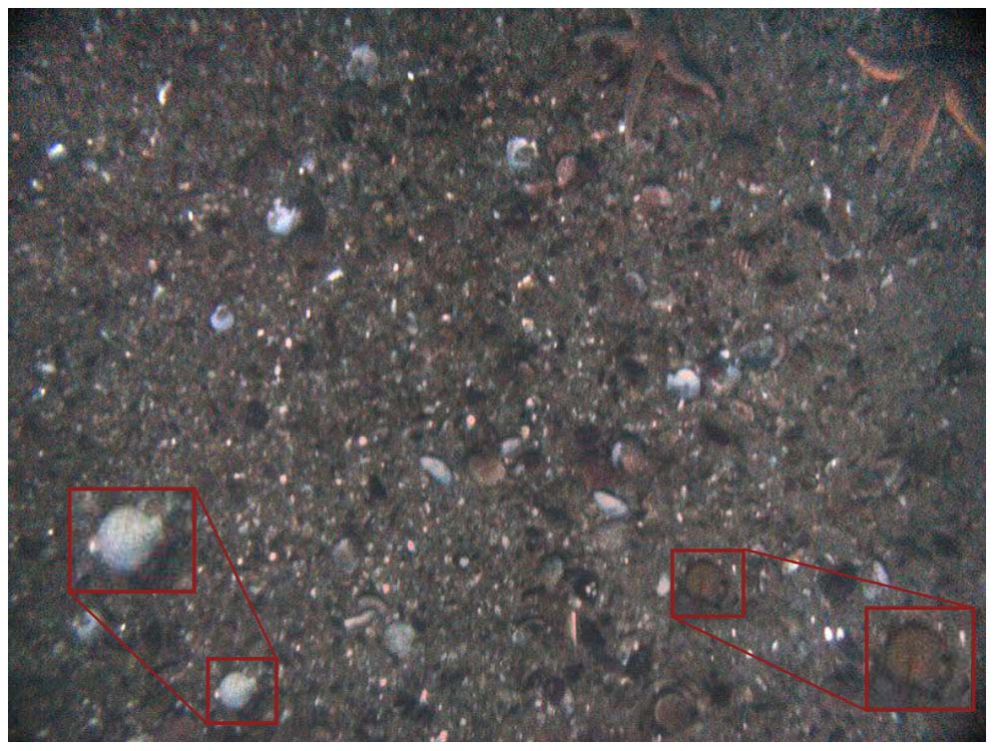
An enhanced color digital image of a scallop bed in Breidafjordur, Iceland, collected from the Gavia autonomous underwater vehicle. The image was taken at 1.96 m above the bottom and 28.9 m depth with a pitch and roll of 0.6 and 2 deg. The bottom right corner shows an enlargement of a live scallop. The bottom left corner shows an enlarged dead scallop shell that has decolorized.

The obtained images were enhanced for color balance and reduction of vignetting effect as described in [11]. Gaussian blur and linear filtering techniques were used to remove noise and sharpen the image. Color contrast was obtained with gamma correction. The image enhancement routine was implemented using EBImage routine in R statistical software (EBImage software version 3.10.0 developed by [20]). The associated metadata on time, latitude, longitude, altitude, depth, pitch, and roll of the vehicle were extracted for each image. Scallops were identified visually from all selected images. The main distinguishing features used for identification were the shape and color of the scallop shell, and the presence of a shadow around the valve (round edge) of the shell. Dead shells tend to break off at the hinges and decolorize. Therefore, scallop shells that had a brighter (whitish) appearance on the images were considered dead (Figure 2). Scallop shell size (height) was measured from the tip of the valve to the umbo (hinge) of the shell where the valves connect. Pixel points 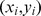 at the tip and the base of each identified shell were recorded for height estimation. Prior to height estimation of objects from underwater photographs, the geometric distortions introduced by the unstable movement of the camera (due to the pitch and roll of the vehicle), and the camera lens need to be corrected [21].

The AUV has a downward facing camera. With the aid of a proper navigation system, which regulates the orientation (pitch, roll, yaw) of the vehicle, the camera takes photographs from the correct angle. This increases reliability in the estimation of the area covered off the seafloor by the survey. At a distance of 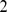 m from the bottom an image covers 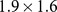 m on the ground [11]. On a 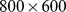 resolution image each pixel represents 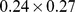 cm on the ground. The effect of pitch and roll on the area covered on the ground was calculated with the use of basic trigonometry, applied to each image. The angle, in deg, between the outside field of view and a straight line (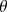) is given by:
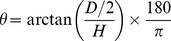
(1)where D represents the horizontal or vertical dimension on the ground and 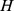 represents the height from the bottom at which the photo was taken.

Any change in the pitch and roll (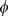) can now be accounted for and a new dimension (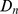) can be calculated by:
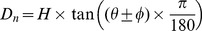
(2)


The average pitch and roll in the dataset were approximately 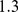 and 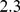 deg, which resulted in an error of approximately 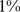 and 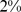 in the estimated horizontal and vertical dimensions of the image. These effects were thus considered negligible. None of the identified scallops were partially in the image therefore accounting for edge effects was not necessary in this analysis.

**Figure 3:**
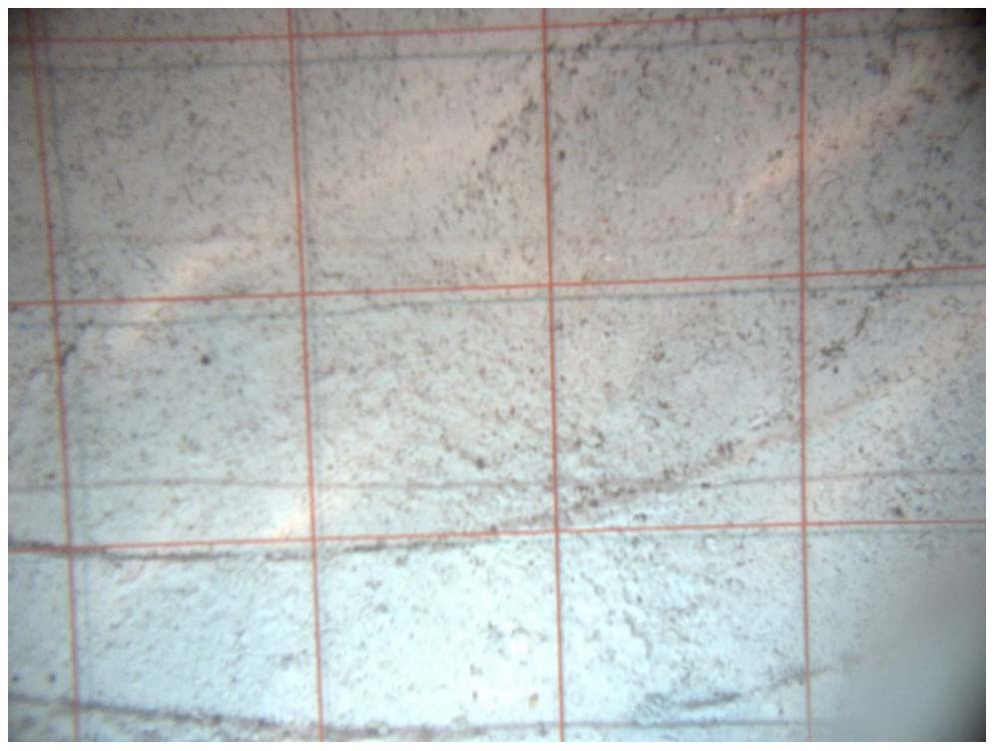
A checkered frame photographed underwater with the AUV camera for lens distortion correction where each square represents 15×15 cm.

A frame with checkered lines was photographed underwater to study the inherent distortions introduced by the camera lens. The frame was photographed in a freshwater pool at a depth of approximately 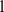 m. A pincushion distortion effect was seen with the lines bending inwards. The bending of the straight lines along the corners indicate that the distortions caused by the camera lens are greater at the edges than the center of the image (Figure 3).

A second-order polynomial regression model was fitted to obtain a lens distortion correction. The identified model successfully predicted a undistorted reference grid (dependent variable) from the distorted matrix (independent variable) extracted from the underwater photo of the frame. The 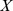 and 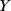 pixel points of the reference grid were modeled separately as follows:
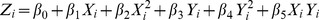
(3)where 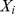 and 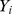 represent the coordinates of the distorted matrix.

**Figure 4:**
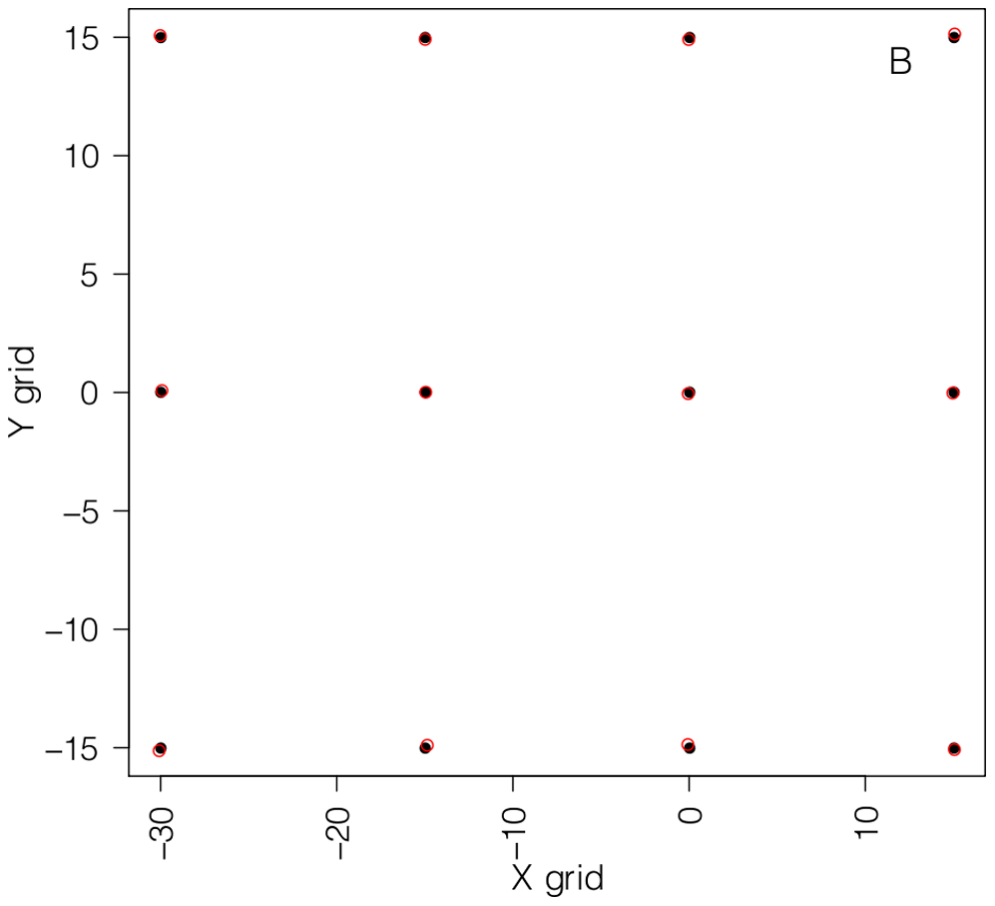
A undistorted reference grid (black) and prediction (red) from the distorted matrix extracted from the image of the checkered frame in **Figure 3**.

The model fit is illustrated in Figure 4 where the fitted values (green) are superimposed on the reference grid. The models were then used to predict the distance 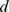 between the pixel points marked at the tip and base of each identified shell. This estimate of 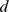 was then scaled for the height from the bottom at which the photo was taken to obtain a size estimate for each identified shell:
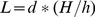
(4)where 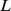 represents the size of the scallop shell, 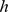 represents the height from the bottom at which the checkered frame was photographed and 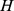 represents the height from the bottom at which the scallop was photographed.

To validate this approach of shell height estimation, a ground-truthing experiment was conducted. Some live scallops were gathered from Breidafjordur, measured and aggregated into the following 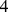 height (cm) categories; 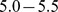, 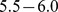, 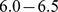 and 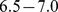. The categories were formed to test whether a difference of 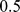 cm in shell height can be detected. The scallops were then placed in separate sections in a net, planted along the intertidal zone, and photographed with the AUV camera at a depth of approximately 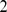 m during high tide. The heights of shells that were lying flat on the seafloor were predicted with the use of the second-order polynomial regression models defined above. These included 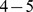 shells per size category.

A nested design random effects model was used to compare the estimated scallop sizes across the 5 repeated surveys:
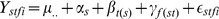
(5)where 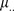 is a constant, 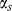 represents the random survey effect, 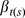 represents the random transect effect nested within survey, 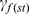 represents the random frame effect nested within the survey and transect, 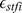 is the random error variable, all with expectations 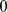 and variances 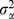, 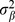, 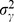 and 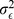 respectively [22].

To determine whether the repeated surveys were significantly different, a full model was compared with a reduced model without the survey effect. The lme4 package [23] in the R statistical software [24] was used for random effects analyses.

### Dredge Survey

The Marine Research Institute of Iceland conducts an annual dredge survey in the inner part of Breidafjordur to monitor the status of the scallop stock. A total of 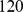 sites are sampled with the use of a 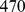 kg sledge dredge of 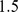 m width. The length of each tow is approximately 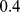 nautical miles. A subsample of scallops are taken from each tow after weighing the total catch. Approximately 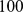 scallops are measured and the remainder are counted [17]. Survey data from September 2011 was used to select a tow that was close to the AUV sampling site. The start and end positions of the tow were 65°05.900′ N 22°43.630′ W and 65°05.660′ N 22°43.010′ W.

A smaller-scale monthly survey is also conducted at 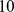 sites where a smaller dredge of size 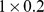 m is used. Dredging is done at a vessel speed of approximately 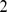 mph with a towing time of approximately 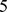 min. Tows are taken at random in the defined site and only the starting point of the tow is recorded. A sample of scallops from each tow are measured and counted. An estimate of the total number of scallops caught in the tow is not calculated. Data from the tow that was in close proximity to the AUV sampling site (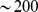 m) was used. The chosen tow was sampled in October 2011. The starting position of the tow was 65°05.557′ N 22°46.643′ W.

## Results

The number of live scallops observed per image ranged from 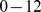. Abundance estimation of scallops from these count data is presented in [11].

**Figure 5:**
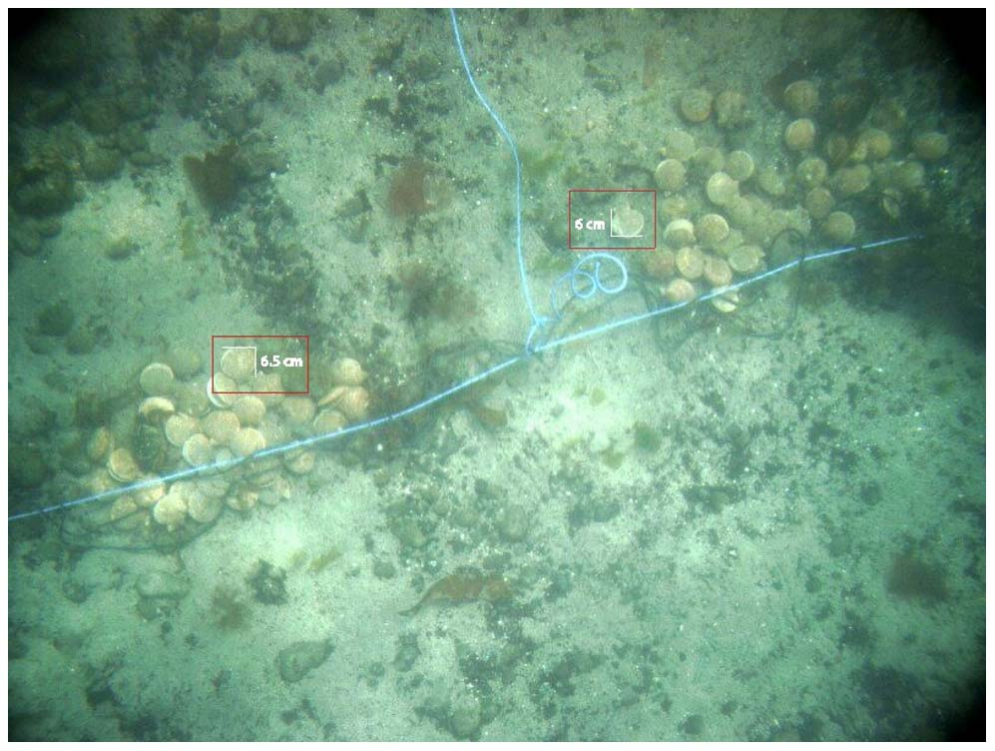
Iceland scallops photographed with a AUV camera at 1.94 m above the bottom, from the water surface. Approximate size scales, 6.0 and 6.5 cm, for known scallop sizes are also displayed.

**Figure 6:**
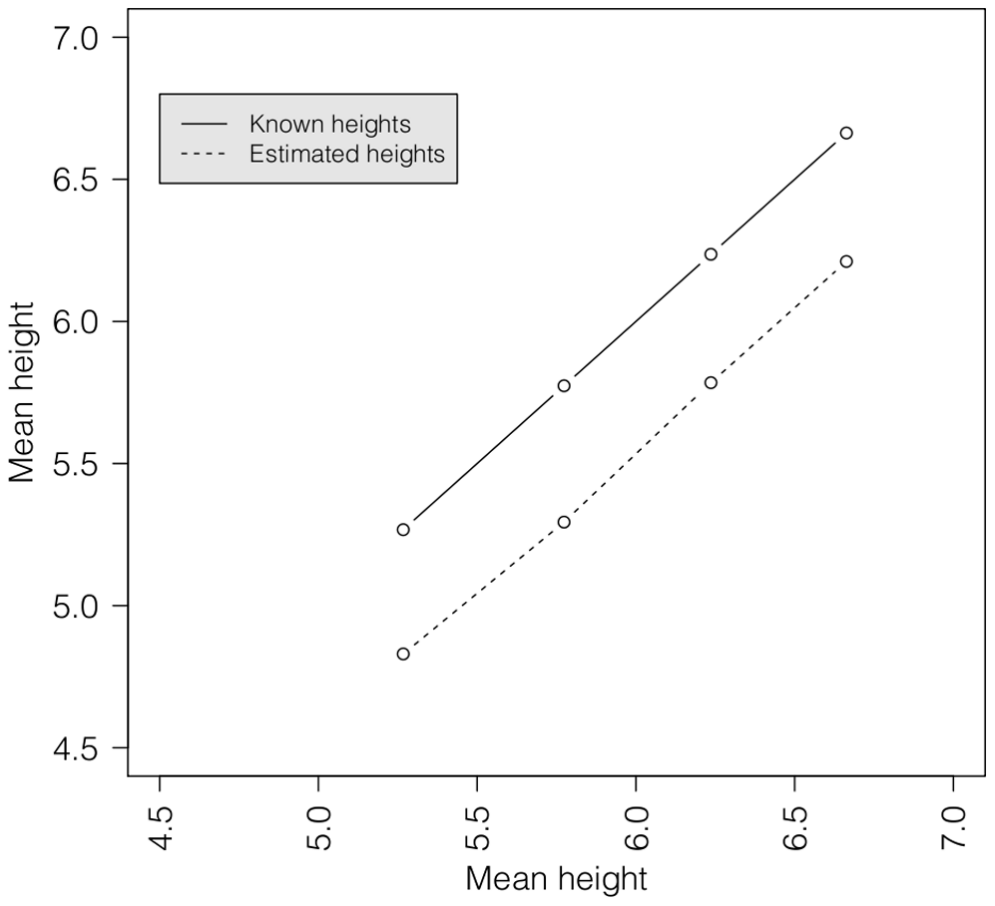
Known and estimated mean heights from 4 size categories of scallops planted along a inter-tidal zone for the ground-truthing experiment.

The images of scallops from the ground-truthing experiment were taken in ambient lighting. These images were thus of a better quality (Figure 5). A comparison of the known and estimated mean heights of the planted scallops showed that the heights were underestimated by approximately 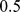 cm in all 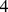 size categories (Figure 6). This could be attributed to pixel error. With each pixel representing 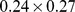 cm, clicking a pixel away on each side could introduce an error of approximately 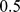 cm. To account for this disparity, a simple linear regression model was used to predict the shell heights based on the relationship between the known and estimated mean shell heights of the planted scallops. The data thus transformed were used in the analysis below.

An analysis of variance between the full model and a reduced model without the survey effect yielded no significant difference (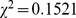) showing that the estimated lengths across the 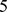 repeated surveys were not significantly different i.e. there is no obvious inconsistency between the AUV surveys.

Models:

lme1: le.dat ^∼^ 1+(1|survey:transect)+(1|survey:transect:frame)

lme2: le.dat ^∼^ 1+(1|survey)+(1|survey:transect)+

(1|survey:transect:frame)

Df AIC BIC logLik Chisq Chi Df Pr(>Chisq)

lme1 4 3758.4 3779.9 −1875.2

lme2 5 3758.4 3785.2 −1874.2 2.0513 1 0.1521

The variability in the data was dominated by the within-frame effect (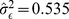) and the frame effect (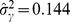). The survey effect and transects within survey effect were not significant (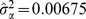, 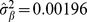).

**Figure 7:**
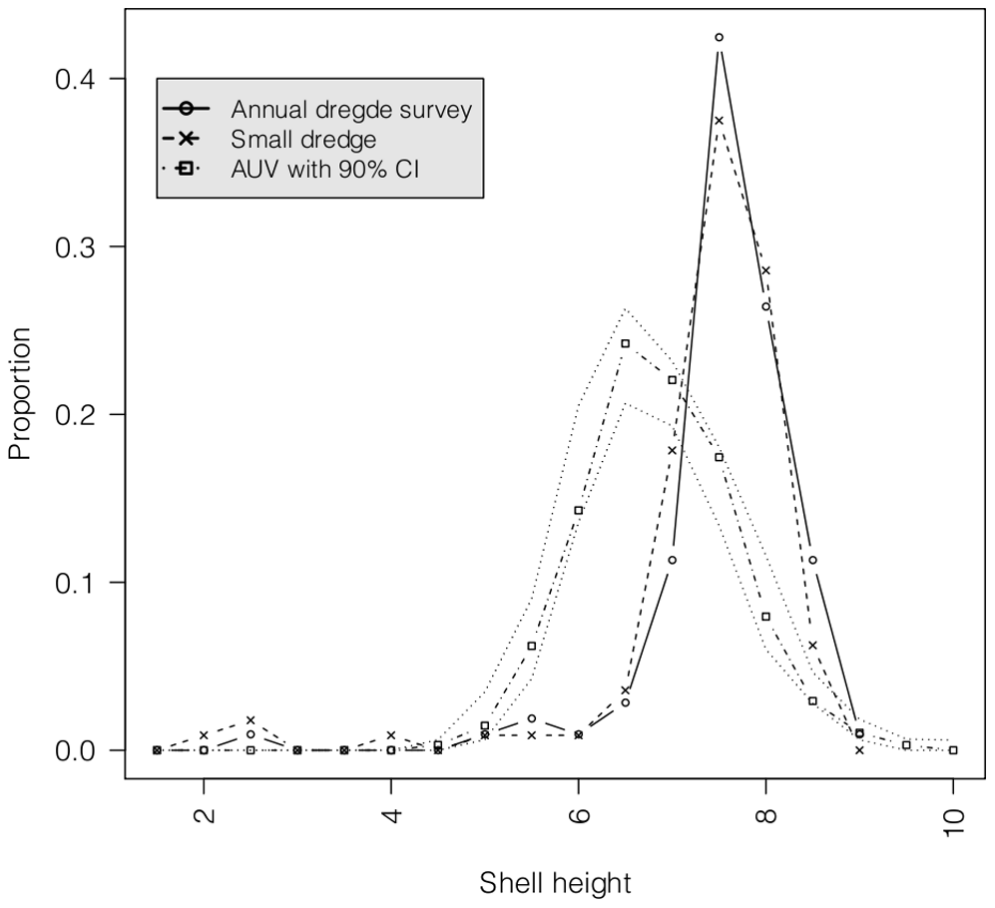
The proportional scallop shell height frequency distribution from the AUV survey with a 90% confidence bound (312 data points), the annual dredge tow (106 data points) and the small-scale dredge tow (112 data points).

The sample size of the 5 AUV surveys ranged from 289–341. The annual dredge tow and the small-scale dredge tow had sample sizes of 106 and 112 respectively. A comparison of the proportional shell height frequency distributions between the AUV and dredge surveys showed non-overlapping peaks (Figure 7). A broader size range of scallops was observed in the AUV survey. The estimated scallop shell heights from the AUV survey ranged from 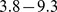 cm with a mean of 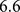 cm. The repeated measurements were used to generate a confidence bound around the shell height distribution obtained from the AUV survey. The mean sizes of scallops observed in the small and annual dredge tows were 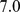 cm and 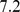 cm.

## Discussion

An efficient survey scheme forms a fundamental component of a good fisheries management system. Improvements in data quality can be accomplished with enhanced data collection techniques and modern technological approaches. In this age of underwater robotics, autonomous underwater vehicles equipped with photographic and acoustic equipment are seen as novel tools that can be used to advance fisheries sampling techniques [10]. The time and cost effectiveness of the AUV survey technique makes it a novel approach, which can be utilized to gather enough data replicates in a given area. This survey was conducted on a small-scale with the aim to establish methodologies and develop the use of the Gavia AUV for management of macrobenthic organisms. In particular, a technique for scallop shell height estimation from AUV photos was presented. An analysis of variance showed that the scallop size estimates across the 5 repeated AUV surveys carried out in the study area were not significantly different. Precision is a desirable factor in marine survey data, and repeated measurements give confidence bounds that can be used to detect modes in size distributions with higher certainty.

Approximately 1 scallop was observed per square meter at the sampled location. The habitat type, as observed from the images, mainly appeared to be gravelly with scattered fragments of dead shells. The observed dominance of larger individuals in the obtained shell height frequency distribution is characteristic of an unfished long-lived species, where larger individuals accumulate in a population over time. The Iceland scallop is a long-lived species that most commonly dwells in waters between 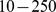 m with a preferred physical environment characterized by strong currents and temperatures between 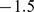 to 8°C [25]. Sexual maturity is related to food availability and surrounding temperature, and the size at maturity is estimated to be around 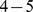 cm shell height at the age of 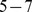 years [18].

Non-overlapping shell height frequency distributions were seen between the AUV and dredge surveys. A narrower size range of scallops was observed in the dredge sample than the AUV sample. This could reflect the size selectivity of a dredge and its bias towards larger individuals in a population, which is a known concern [3], [7]. The dredge used for scallop surveys contains rings of approximately 6 cm in diameter. Hence, larger individuals dominate the height frequency distribution. Further, the tow from the annual dredge survey used for comparison was approximately 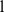 km away from the AUV survey location and falls in a slightly deeper area (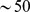 m) than the AUV survey. Scallop distribution is known to be correlated with water depth with a shift toward the deeper waters with increasing size [26]. It could be possible that bigger scallops were present in this deeper area. It was hard to determine the direction of the small-scale dredge tow as only the starting point of the survey was known. However, it was nearer to the AUV sampling site than the annual survey tow. It is well-known that standard tests comparing such frequency distributions can not be used due to the correlations in the data [27], but the difference here is compelling since the AUV finds many more scallops of smaller sizes. The outcome that the AUV finds scallops of a much wider size range highlights the bias of the dredge survey.

On the other hand, shell heights from the AUV survey could potentially be underestimated due to the low resolution of the photographs, e.g. due to pixel error. The edges of the shells appeared fuzzy and hence were difficult to detect with certainty. This pixel error could also be defined as operator error if the operator had the tendency to click on the inner edges of the shells. Only one operator identified and marked the scallops. Although, the model used for shell height estimation was validated with ground-truthing data on planted scallops of known sizes, the quality of the photos used for validation was much higher than the images from the actual survey. These calibration photos were taken in ambient lighting whereas the survey was conducted in low light and murky conditions. Additionally, identification of the shells in the survey was sometimes challenging due to the low resolution of the photos. The orientation of the shells could also be disputed at times. This would lead to the diameter of the shell being estimated instead of the height, a challenge also faced by [6].

The low resolution images made identification of smaller scallops difficult. Some small scallops were observed in the dredge survey. These could be juvenile scallops that tend to attach themselves to the inner part of the older shells and get collected in the dredge [28]. Overall, the observations of very few small scallops in both surveys could be attributed to low recruitment levels in the recent years due to the known scallop parasite outbreak in the region. Starfish *Asterias rubens*, which are the chief predators of scallops were also observed on a number of images where scallops were found. These predators are known to feed on juvenile scallops [28].

Basic image enhancement techniques were used here to improve the image quality; however, investigations into a better camera system are underway. Some of the problems faced here can be alleviated with higher resolution digital photos, but there will always be a need for image enhancement because a better camera will merely move the limits of detection towards higher altitudes (distance from the bottom at which photos are taken) and smaller organisms. The ground-truthing experiment showed that it was possible to detect a difference of 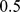 cm in shell heights. For the same altitude, this can be improved with better image quality. Given that it was seen that the vehicle position has some uncertainty, a better navigation system would also be desirable.

A small-scale comparison was made between the AUV and dredge survey data; however further investigations such as that carried out by [29] are necessary to evaluate any underlying bias and to validate how representative these surveys are of the true population. The preliminary results of this study, however, indicate that the AUV might provide a better estimate of population structure because a broader size range of scallops were seen. Nevertheless, a more detailed comparison with an improved camera system is needed prior to any firm conclusions.
